# Histone H3 lysine-trimethylation markers are decreased by recombinant methioninase and increased by methotrexate at concentrations which inhibit methionine-addicted osteosarcoma cell proliferation

**DOI:** 10.1016/j.bbrep.2021.101177

**Published:** 2021-11-26

**Authors:** Yusuke Aoki, Yasunori Tome, Qinghong Han, Jun Yamamoto, Kazuyuki Hamada, Noriyuki Masaki, Michael Bouvet, Kotaro Nishida, Robert M. Hoffman

**Affiliations:** a AntiCancer Inc, 7917 Ostrow St, San Diego, CA, 92111, USA; b Department of Surgery, University of California, San Diego, 9300 Campus Point Drive #7220, La Jolla, CA, 92037-7220, USA; c Department of Orthopedic Surgery, Graduate School of Medicine, University of the Ryukyus, 207 Uehara, Nishihara, Okinawa, 903-0125, Japan

**Keywords:** Osteosarcoma, Recombinant methioninase, Methionine restriction, Methotrexate, Methylation, Histone, H3K9me3, H3K27me3

## Abstract

Methionine addiction is a fundamental and general hallmark of cancer cells, which require exogenous methionine, despite their ability to synthesize normal amounts of methionine from homocysteine. In contrast, methionine-independent normal cells do not require exogenous methionine in the presence of a methionine precursor. The methionine addiction of cancer cells is due to excess transmethylation reactions. We have previously shown that histone H3 lysine marks are over-methylated in cancer cells and the over-methylation is unstable when the cancer cells are restricted of methionine. In the present study, we show that methionine-addicted osteosarcoma cells are sensitive to both methotrexate (MTX) and recombinant methioninase (rMETase), but they affect histone H3 lysine-methylation in the opposite direction. Concentrations of MTX and rMETase, which inhibit osteosarcoma cells viability to 20%, had opposing effects on the status of histone methylation of H3K9me3 and H3K27me3. rMETase significantly decreased the amount of H3K9me3 and H3K27me3. In contrast, MTX significantly increased the amount of H3K9me and H3K27me3. The results suggest that increase or decrease in these methylated histone lysine marks is associated with proliferation arrest of methionine-addicted osteosarcoma.

## Highlights

• Osteosarcoma cells are sensitive to both methotrexate and recombinant methioninase.
• MTX increased the amount of H3K9me and H3K27me3.
• RMETase decreased the amount of H3K9me3 and H3K27me3.
• Increase/decrease in H3K9me3 and H3K27me3 is associated with proliferation arrest.

## 1 Introduction

Methionine addiction, a general and fundamental hallmark of cancer, was discovered by one of us (Robert M. Hoffman) [1]. Methionine addiction is due to overuse of methionine for transmethylation reactions [2]. At least some of the overused methionine by methionine-addicted cancer cells is used for over-methylation of histone H3 lysine marks [3].

In addition, we previously have reported that the levels of histone H3 lysine-trimethylation were unstable in methionine-addicted cancer cells under methionine-restriction (MR) conditions [3], effected by recombinant l-methionine α-deamino-γ-mercapto-methane lyase (rMETase) [4]. In contrast, normal cells have a stable and normal level of histone H3 lysine-trimethylation, even under MR [3].

Methotrexate (MTX) is a first line therapy for osteosarcoma, which has been used for decades [5,6]. MTX inhibits dihydrofolate reductase, subsequently depleting 10-formyl-tetrahydrofolate, which is essential for *de novo* purine synthesis, and 5,10-methylente-tetrahydrofolate, which is used for pyrimidine synthesis, causing inhibition of DNA synthesis.

The global status of the histone lysine-methylation may alter the expression of a vast number of genes [[7], [8], [9]]. Histone H3K9me3 and H3K27me3 are thought to be involved in the repression the gene transcription. We previously reported that the levels of the histone H3K9me3 and H3K27me3 were decreased in methionine-addicted cancer cells when treated with rMETase [3,10]. There are previous reports on the relationship of drug resistance and the levels of H3K9me3 and H3K27me3, but they are not consistent [[11], [12], [13], [14], [15], [16], [17], [18], [19], [20], [21], [22]]. There are no reports on the effect of MTX on histone H3 lysine-methylation.

In the present report, we show that rMETase and MTX have opposite effects on histone H3 lysine-methylation, at concentrations which inhibit proliferation of methionine-addicted osteosarcoma cells.

## 2 Materials and methods

### 2.1 Cell culture and reagents

The 143B human osteosarcoma cell line was obtained from the American Type Culture Collection (Manassas, VA, USA). Cells were cultured in Dulbecco's Modified Eagle Medium (DMEM) supplemented with 10% fetal bovine serum (FBS) and 1 IU/ml penicillin/streptomycin.

### 2.2 Reagents

MTX was obtained from MedChemExpress (# HY-14519, Monmouth Junction, NJ, USA) and dissolved in dimethyl sulfoxide (DMSO). Recombinant methioninase (rMETase) (AntiCancer Inc., San Diego, CA, USA) is a homotetrameric enzyme, with a 172-kDa molecular mass. Production of rMETase was as previously reported [4].

### 2.3 Drug sensitivity assay

Cell viability was assessed using the WST-8 reagent (Dojindo Laboratory, Kumamoto, Japan). Cells were cultured in 96-well plates (7.5 × 10^2^ cells/well) in DMEM (100 μl/well) and incubated at 37 °C overnight. Cells were treated with different concentrations of MTX, between 1.25 μM and 160 μM; or rMETase, between 0.025 U/ml and 1.6 U/ml for 72h. At the end of the culture period, 10 μl of the WST-8 solution was added to each well and the plate was additionally incubated for 1 h at 37 °C. Absorption was measured with a microplate reader (SUNRISE: TECAN, Männedorf, Switzerland) at 450 nm. Drug sensitivity curves were obtained with Microsoft Excel for Mac 2016 ver. 15.52 (Microsoft, Redmond, WA, USA) and half-maximal inhibitory concentration (IC_50_) values were calculated with ImageJ ver. 1.53k (National Institutes of Health, Bethesda, MD, USA). Experiments were performed twice, each in triplicate.

### 2.4 Immunoblotting

The cells were cultured in 100 mm^2^ dishes in DMEM medium, incubated in 37 °C overnight. The dishes were washed with phosphate-buffered saline (PBS) one time, and medium was changed to normal medium or medium with MTX (0.02 μM) or with METase (0.5 U/ml) and further incubated in 37 °C for 72h. The cells were lysed, and histones were extracted using a Epiquik Total Histone-Extraction Kit (Epigentek, Farmingdale, NY, USA). Immunoblotting for these histones was performed as follows: Histone extract samples were loaded onto 12% SDS-PAGE gels and transferred to 0.2 μm polyvinylidene difluoride (PVDF) membranes. Blocking of the membranes was performed with the Bullet Blocking One for Western Blotting (Nakalai Tesque, Inc. Kyoto, Japan). *Anti*-H3K9me3 antibody (1:1,000, #13969, Cell Signaling Technology, Danvers, MA, USA); *anti*-H3K27me3 antibody (1:1000 #9733, Cell Signaling Technology); and *anti*-H3 antibody (1:1,500, 17168-1-AP, Proteintech, Rosemont, IL, USA) were used. Total histone H3 was used as an internal loading control. Horseradish-peroxidase-conjugated *anti*-rabbit IgG (1:20,000, SA00001-2, Proteintech, Rosemont, IL, USA) was used as a second antibody. Immunoreactivity was visualized with Clarity Western ECL Substrate (Bio-Rad Laboratories, Hercules, CA, USA). The signals were detected with the UVP ChemStudio (Analytik Jena, Upland, CA, USA) [3,10]. The signals of H3K9me3 and H3K27me3 were normalized to the signals of total histone H3 for relative quantification. Experiments were performed three times.

### 2.5 Statistical analysis

All statistical analyses were performed with JMP pro ver. 15.0.0 (SAS Institute, Cary, NC, USA). Tukey-Kramer HSD was performed to compare the means between 3 groups. The Dunnett test was applied to compare each of the means with the control group. Bar graphs were constructed to show the mean and error bars show standard deviation or standard error of the mean. A probability value ≤ 0.05 was defined as statistically significant.

## 3 Results

### 3.1 Osteosarcoma cells are sensitive to both rMETase and MTX

**Fig. 1:**
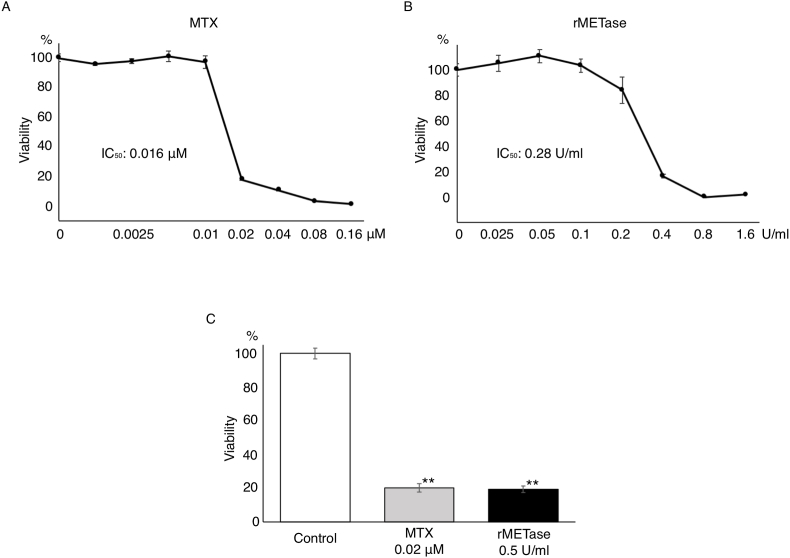
MTX and rMETase sensitivity of human osteosarcoma cell line 143B (mean ± SD, n = 3). (A) Sensitivity to MTX. (B) Sensitivity to rMETase. (C) Comparison of the efficacy of MTX (0.02 μM) and rMETase (0.5 U/ml). **; P < 0.001.

Both MTX and rMETase inhibited the 143B osteosarcoma cells with the following IC_50_: MTX (0.016 μM), and rMETase (0.28 U/ml) (Fig. 1A and B), (MTX [P < 0.001], and rMETase [P < 0.001], compared to the untreated controls) (Fig. 1C).

### 3.2 rMTEase decreased and MTX increased histone H3 lysine-trimethylation

**Fig. 2:**
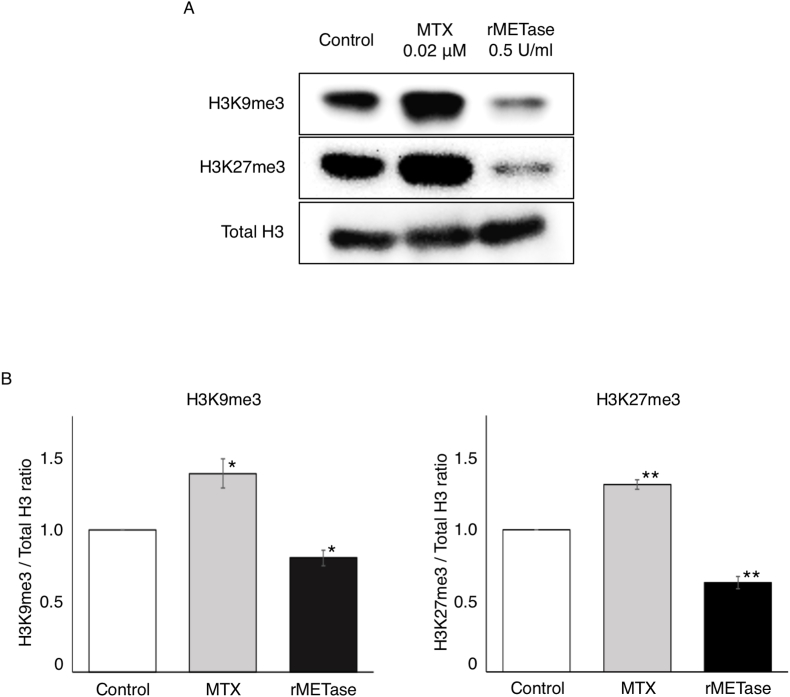
(A) Immunoblotting of histone H3 lysine marks H3K9me3 and H3K27me3 in 143B osteosarcoma cells cultured in DMEM or treated with MTX (0.02 μM) or rMETase (0.5 U/ml) for 72h. (B) The ratio of H3K9me3/total histone H3 and H3K27me3/total histone H3 in 143B osteosarcoma cells cultured in DMEM and treated with MTX or rMETase (mean ± SEM, n = 3). *; P < 0.01, **; P < 0.001.

We evaluated the methylation status of histone H3K9me3 and H3K27me3 in osteosarcoma cells by immunoblotting, at the 20% inhibitory concentrations of rMETase or MTX. Compared to untreated control cells, rMETase decreased the amount of H3K9me3 (P = 0.04) and H3K27me3 (P < 0.001). In contrast, MTX increased the amount of H3K9me3 (P = 0.002) and H3K27me3 (P < 0.001) (Fig. 2).

## 4 Discussion

The results of the present study show that the levels of the histone H3 lysine marks H3K9me3 and H3K27me3 were decreased in methionine-addicted osteosarcoma cells treated with rMETase, which is consistent with our previous study, which showed that the level of H3K9me3 and H3K27me3 was decreased in methionine-addicted carcinoma cells by treatment with rMETase [3,10]. In contrast in the present study, the level of H3K9me3 and H3K27me3 was increased by MTX.

There are reported effects of drugs on histone H3 lysine-methylation, including H3K9me3 and H3K27me3 [[11], [12], [13], [14], [15], [16], [17], [18], [19], [20], [21], [22]], which are inconsistent. There are no reports on the effect of MTX on histone-H3 lysine-methylation. The present report is the first to show histone H3 lysine-trimethylation marks are increased by MTX.

In the present study, we compared the status of histone H3 lysine-trimethylation between cells treated with rMETase and MTX, and found an opposite effect with these 2 agents. Generally histone H3K9me3 and H3K27me3 are regarded as transcription suppressors [[7], [8], [9]]. The global status of histone methylation might alter the expression of a vast number of genes. The results suggest that both an increase and a decrease of histone H3 lysine-methylation can be involved in inhibition of proliferation of methionine-addicted cancer cells.

MTX is a long-time first-line drug for all sarcoma types. rMETase has shown efficacy against all major sarcoma types in patient-derived orthotopic xenograft (PDOX) mouse models [[23], [24], [25], [26], [27], [28], [29], [30], [31], [32], [33], [34], [35], [36]], and has shown clinical efficacy as well in preliminary studies [37].

rMETase reduces the cell's external source of methionine. MTX reduces the cell's internal source of methionine by inhibiting dihydrofolate reductase (DHFR) and methylenetetrahydrofolate reductase (MTHFR), thereby inhibiting methionine biosynthesis from homocysteine. Our previous studies have indicated that cancer cells use external methionine and biosynthesized methionine differently [1,38], which appear to be in separate pools.

Our current hypothesis is that the inhibition of internally-biosynthesized methionine by MTX may disrupt the methionine balance in the cancer cell, possibly allowing more external methionine to be used for histone methylation via S-adenosylmethionine (SAM). It is possible that both over-methylation and under-methylation of the two histone markers, H3K9me3 and H3K27me3, are detrimental to the proliferation of osteosarcoma cells.

Future studies will focus on the mechanism of cell proliferation inhibition by rMETase and MTX, involving alteration of histone H3 lysine-methylation, including studies at the atomic/molecular level [39,40].

## Declaration of competing interest

The authors declare that they have no known competing financial interests or personal relationships that could have appeared to influence the work reported in this paper.

## Data Availability

Data will be made available on request.
